# Lightweight Adaptation of Classifiers to Users and Contexts: Trends of the Emerging Domain

**DOI:** 10.1155/2015/434826

**Published:** 2015-09-10

**Authors:** Elena Vildjiounaite, Georgy Gimel'farb, Vesa Kyllönen, Johannes Peltola

**Affiliations:** ^1^VTT Technical Research Centre of Finland, Kaitoväylä 1, 90571 Oulu, Finland; ^2^The University of Auckland, Private Bag 92019, Auckland 1149, New Zealand

## Abstract

Intelligent computer applications need to adapt their behaviour to contexts and users, but conventional classifier adaptation methods require long data collection and/or training times. Therefore classifier adaptation is often performed as follows: at design time application developers define typical usage contexts and provide reasoning models for each of these contexts, and then at runtime an appropriate model is selected from available ones. Typically, definition of usage contexts and reasoning models heavily relies on domain knowledge. However, in practice many applications are used in so diverse situations that no developer can predict them all and collect for each situation adequate training and test databases. Such applications have to adapt to a new user or unknown context at runtime just from interaction with the user, preferably in fairly *lightweight* ways, that is, requiring limited user effort to collect training data and limited time of performing the adaptation. This paper analyses adaptation trends in several emerging domains and outlines promising ideas, proposed for making multimodal classifiers user-specific and context-specific without significant user efforts, detailed domain knowledge, and/or complete retraining of the classifiers. Based on this analysis, this paper identifies important application characteristics and presents guidelines to consider these characteristics in adaptation design.

## 1. Introduction

As was observed in [1], conventional data mining is driven by academic interests (e.g., the development of innovative algorithms) rather than by practical considerations. At the same time its applications need to account for the whole process of solving real-world problems, including user interactions and influence of environmental factors. Just the same holds for classification: for example, research into classification methods for pervasive computing largely focused on datasets, collected in research labs or environments, occupied by researchers, and ignored diversity of real-world settings [2]. Systems' evaluation should be also more realistic; for example, not only accuracy should be assessed but also the amount of efforts/resources, spent to achieve this accuracy, should be measured [3].

This paper focuses on practical solutions for runtime situational adaptation of classifiers in cases, where context-independent reasoning either notably decreases the system accuracy or causes considerable users' discontent. Multiple existing surveys of reasoning methods in various research and application areas, for example, [4–7], to cite only a few most recent ones, either do not discuss context adaptation at all or do not distinguish lightweight context adaptation methods from less practical ones. Contrastingly, our review below focuses on techniques, proposed for adaptation to large varieties of users and contexts and requiring neither significant explicit interaction efforts nor detailed domain knowledge. We analyse influence of various context types on classification methods and suggest which adaptation techniques better suit different types of changes in user and system behaviour. The obtained recommendations can be beneficial for designing adaptive systems in frequent practical cases where the successful adaptation could be notably helpful for the users, while the unsuccessful one would not cause serious problems.

The paper is organised as follows.  Section 2 discusses limitations of the conventional adaptation approaches; Section 3 presents an overview of the lightweight adaptation; and Section 4 details and compares approaches, proposed in the selected research areas. Recommendations on designing the adaptation and concluding remarks are presented in Sections 5 and 6, respectively.

## 2. Limitations of Conventional Adaptation Approaches

The majority of classification systems do not provide for diversity of real-life situations. Often, classifiers are developed for the only usage context and heavily rely on knowledge about specifics of this target context, acquired ad hoc from an expert and applied to solutions that can hardly adapt to new conditions [7]. For example, event detection in multimedia analysis systems is often based on recognising small sets of context-specific sounds or visual objects [8–10] and hence reducing dependency of multimedia analysis systems on domain knowledge is a serious challenge [11, 12].

Most common approach to context adaptation is to specify typical situations at design time, to develop a separate classification model for each of these situations, and to define mappings between the situations and corresponding classification models. Then at runtime these mappings are used for model selection. For example, affect recognition systems utilised separate classification models for females and males [13, 14] or for silent and talking users [15], whereas recommender systems employed different reasoning strategies for heterogeneous and homogeneous groups [16, 17]. Similarly, a physical activity recognition system in [18] recognised eight predefined contexts and then selected classification models to recognise sets of context-specific activities: for example, “typing”, “sitting,” and other activities in a “lab” context were recognised by one set of classifiers, while “sleeping,” “eating,” “sitting,” and other activities in “home” context were recognised by a different classifier set. However, this approach allows only a coarse adaptation and fails in contexts which were not predefined (e.g., real life is not limited to the eight contexts, selected in [18]) and when humans do not behave according to expectations of system developers (as is often the case); for example, emotion expression of a reserve female may be closer to males' ways, and activities may be not so strictly linked to places (e.g., “typing” activity may occur not only in a “lab” context if a person often works at home, in airplanes, etc.).

Accounting for personal differences, social rules, and etiquette is said to be an important goal for recommender systems [19, 20], interactive systems [21, 22], and affect recognisers [23]. Adaptation of information retrieval systems to personal differences has also gained more attention recently [24]. But social rules and personal differences are not strict, are difficult to formulate, and vary significantly depending on person's culture and age. Other examples of elusive contexts are personal goals (e.g., search intents) and variations between environments. Due to the latter, adapting the model to new scenes is listed among the most significant challenges for intelligent environments [2, 25]. As it is virtually impossible for application developers to predefine all usage contexts of their applications [22], adaptation should be performed at runtime using context-specific data.

Recently, involvement of the end users into runtime adaptation process has gained importance [4, 22]. Unfortunately, conventional learning methods do not suit well this purpose. Conventional supervised learning methods require too large datasets for each context to acquire from end user [26], whereas conventional unsupervised and semisupervised methods often employ domain knowledge-based assumptions, which may not hold in all contexts [26]. Furthermore, unsupervised and semisupervised learning schemes favour statistically dominant patterns and thus cannot adapt easily to peculiarities of elusive contexts.

Therefore the runtime context adaptation requires developing methods, capable of learning from small amounts of explicitly and/or implicitly labelled data. If available, unlabelled data shall be also utilised. Recently, such novel techniques were proposed in several application domains: multimedia analysis and retrieval, recommender systems, emotion recognition, and user interaction, but understanding and exploitation of context in fusion systems are still very limited [7]. Below we will describe the concept of lightweight runtime adaptation and summarise suitable approaches.

## 3. Overview of Lightweight Situational Adaptation Techniques

Situational adaptation to a new context can take two forms: (1) training a model from scratch and (2) modifying a model, which has been already trained on one or more other contexts. In both cases, the adaptation is* lightweight* if its costs are considerably lower than for conventional training of the same application and therefore are acceptable for the end user. The costs include data collection, annotation, and reasoning-related computations. This definition is necessarily informal, because the concept of  “user acceptance” hardly can be quantified: it depends on the user's personality, perceived benefits of using the application, convenience of the user-application interaction, and many other nonquantitative psychological factors.

Most of the present systems need large training datasets and intensive computations in order to complete the training process, that is, to estimate all model parameters. Conventional adaptation approaches reduce the need in explicit interaction efforts at the cost of increased need in computational resources: for example, conventional unsupervised and semisupervised learning methods are computationally demanding. The lightweight adaptation of practical interest should rely on a limited user's feedback about the ongoing classification and (possibly) on data of other contexts, to which the system was previously adapted. Therefore the lightweight adaptation should rely on limited modifications rather than total retraining of classifiers; for example, model parameters can be estimated incrementally or partially in order to use the available training data most efficiently. Therefore lightweight adaptation solutions require significantly less explicit interaction efforts than conventional approaches without the need in extra computational resources; most often, lightweight approaches require notably less computational resources than conventional ones.

To the best of our knowledge, research into adaptation has not yet provided guidance on choosing trade-offs between the adaptation costs and achieved accuracies. For example, works studying adaptation of algorithm granularity (such as finer or coarser data clustering) to computational resources and users' needs [27, 28] did not provide guidelines on choosing the adaptation parameters. Works, suggesting that system evaluation should include assessment of efforts, required for achieving system goals [3], did not propose trade-offs either. Furthermore, perception of data labelling difficulty depends on a person [29], and user satisfaction with the algorithm's accuracy depends on many factors, including personal users' attitudes towards adaptation and screen size [30, 31]. For example, even as weak as 50% accurate predictions of user interface (UI) preferences are already beneficial for the users of small devices, whereas for larger screens higher prediction accuracy is required to satisfy the users [30]. Thus we suggest choosing the adaptation granularity as follows: employ first, whenever feasible, the lightweight adaptation and perform the conventional finer one either when the lightweight method fails or during the application idle times if the data necessary for adaptation can be collected with no or only minor (nonannoying) explicit user feedback. But detecting the adaptation failure should rely on the user feedback due to differences in the users' needs and attitudes.

This paper focuses on adaptation of classifiers, combining multimodal data via class- or decision-level fusion, because the adaptation of feature-level fusion is more computationally and data demanding. Inputs to these fusion models, called below “cues,” can be context descriptors, audio classes, levels of optical flow, interaction modalities, TV programme metadata, and so forth. These cues are provided by just the same lower-level models in all situations. Usually, it is assumed that the lower-level models were built at design time using a sufficiently large development dataset, collected for a context, somewhat similar to the application usage contexts with respect to the cue types and their statistical properties. Types of the cues in the suggested adaptation approaches should be predefined at design time, but exact sets of the cues should not necessarily be predefined: it depends on the chosen reasoning methods. Such a multimodal fusion adaptation may fail if the context change causes poor performance of the low-level models. On the other hand, when adaptation cannot be performed on all levels (e.g., due to insufficient amount of training data), updating top levels is more efficient than updating the lower ones [32]. Also, it is easier for the users to provide explicit feedback on the final classification result, and this feedback is more reliable than the feedback on outputs of lower-level models because these outputs may be unclear to the users or perceived as irrelevant. Employing the feedback on the classification results for updating the lower-level models is not an easy matter as well, due to its nonstraightforward propagation to the lower levels: for example, different modalities may not contribute to the final result in the same way in different contexts.

### 3.1. Context Types

The terms “situation” and “context” are often used interchangeably; these terms refer to some kind of external or latent factors influencing users' or system's behaviour. In particular, these terms may specify not only fine descriptors, such as time and noise, but also higher-level abstractions like events, locations, names of databases, and so forth. Most often the classifiers are adapted to the context types representing historical, social, task, environmental, and computational factors.

- Historical factors embrace anything in the past that may affect current state, for example, user or system actions, changes in user's mood or appearance over time, and recently viewed movies.
- Social factors include rules and customs of interaction between humans, for example, gender/age-dependent behaviour and what is considered polite in different situations.
- Task factors present specific users' objectives, for example, purpose of information search and available time.
- Environmental factors are anything in surroundings that may affect sensor readings, for example, background noise and light.
- Computational factors specify system settings, for example, availability or quality of a certain data type (such as image resolution), computational power, and algorithm capabilities.

The most popular context model, called a “representational view” [33], describes the context by a set of features, defined at design time. An alternative more difficult and less common “interactional view” [33] assumes that the context cannot be described with a predefined feature set. Instead, the scope of descriptors is defined dynamically during human activities.

**Figure 1:**
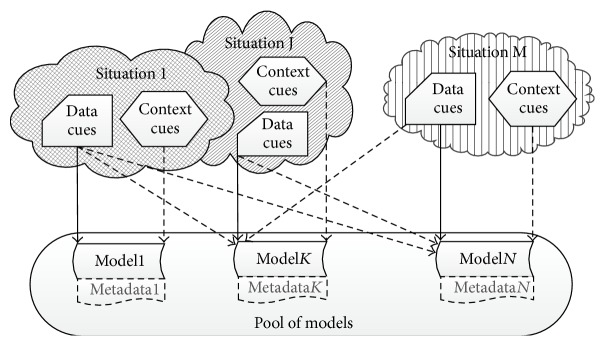
Modelling situation-dependency scenario; dashed lines denote optional data.

The lightweight runtime adaptation to previously unseen contexts typically employs a mixed model in Figure 1: the fine descriptors (“context cues” in Figure 1) or their types are predefined at design time, whereas the higher-level ones (the “situations” in Figure 1) are defined dynamically at runtime. Dynamic definition of high-level contexts can be achieved via analysis of primary data, for example, segmentation [34] or matrix factorisation [35]. The context change can be also detected via analysis of external factors, for example, context features or user interaction (e.g., users may explicitly declare the change by naming a new context or implicitly indicate it by correcting classification errors and requesting adaptation). Below we assume that analysis of external factors was employed.

Most often, the contextual factors (especially, the high-level ones) are nondiscriminative with respect to a classification task at hand; that is, they do not directly help to classify data; rather, situational changes cause certain changes in primary data. In some cases context may influence the user behaviour: for example, humans usually are freer in expressing feelings when conversing with close friends than with officials. In other cases context may influence internal system functionality: for example, success of audio/visual analysis depends on background. Changes in the primary data cues can be categorised as follows:

- *Meaning Changes*. Just the same input cues have to be interpreted differently in different contexts. Often, meaning of cues depends on social factors: for example, whistling that indicates game highlight in basketball matches is a meaningless sound in tennis [8]. Meaning of the cues may depend on historical factors, too (e.g., the users' laughter during a dialog may be interpreted as either happy or sarcastic, depending on the previous statements).
- *Influence Changes*. Importance of the same input cues may vary in different contexts (often, also due to social factors): for example, presence of young children may strongly affect the choice of TV programmes to watch in one family, whereas adults may dominate in another family. Task factors play this role, too: for example, noise or illumination cues may be more or less important, depending on a search goal.
- *Accuracy Changes*. The same input cues may be recognised more or less reliably in different contexts, often due to computational factors; for example, the accuracy of image analysis depends on image resolution. When sensor data are collected in uncontrolled conditions, the environmental factors significantly influence the accuracy of input cues: for example, image background affects the accuracy of object detection. Historical factors may degrade the accuracies, too, when the models become outdated; for example, growing hair may decrease the face recognition accuracy.
- *Availability Changes*. The same input cues may be abundant in some situations and missing in others. Most often this happens in uncontrolled conditions: for example, results of video analysis may be unavailable if users bypass a camera. Social factors may cause incomplete data, too: if it is polite to stay silent, audio cues will be unavailable.

### 3.2. Typical Adaptation Goals, Data Acquisition, and Reasoning Methods

**Multimedia analysis and retrieval systems** aim at detecting various events or concepts, usually, by trained classifiers. An overview of conventional reasoning methods in this area can be found in the earlier surveys [9, 11, 24, 36]. The detection has to be adapted to both task and computational contexts, that is, to different user queries and different multimedia databases, respectively. Differences between the multimedia databases present a challenge because the algorithms, trained on one multimedia type (e.g., genre) or source (TV channel) and tested on another type/source, are usually 1.5–2 times less accurate than the corresponding within-type/source ones [37, 38]. The inter-user differences present an additional challenge: the adaptation to user queries has to be very quick. Therefore, the retrieval systems are often hierarchical: their lower layers perform context- and user-independent multimedia analysis, and their upper ones are iteratively adapted by reranking results of the lower layers [36]. Event and concept detection also have to be adapted to environmental context because they significantly depend on the background.

All these contexts are difficult to predefine. Therefore in this research area several methods to use fairly small datasets either for runtime training of models from scratch or for knowledge transfer between contexts were proposed. Often, explicit data is collected by asking the users to select which items to annotate and/or to correct system outputs. Hence obtained explicit data may be noisy, but an alternative approach, to provide labels on system-selected items from scratch, is more tiresome and may give more than 10% of errors [39]. Investigation of implicit information gathering approaches gained more attention recently [24], but implicit feedback is less reliable than explicit one because clicked results are not always relevant to the user's search [24]. Noise in datasets, however, is most often dealt with in a fairly straightforward way: by employing generic noise-tolerant classifiers, such as support vector machines (SVMs) [36, 40].

**Recommender systems** aim at finding items, most interesting for the target users in the target context. An overview of conventional reasoning methods in this area can be found in the earlier surveys [19, 41, 42]. Mostly, the recommenders adapt to the differences between users' personalities, to influence of context on interests of individuals, and to social context, but usually these contexts have to be predefined: for example, the adaptation to the user's goal (task context) is often performed by predefining a few typical contexts (e.g., watching a movie at home versus in cinema). Many recommender systems employ collaborative filtering (CF), a lazy reasoning method, based on the assumption that users, similar in the past, will also remain similar to some extent in future. Accordingly, the CF searches for users, similar to the target user, and creates recommendations for the target user by combining choices of similar users. Frequently, the CF adapts to contexts by so-called “pre-filtering” [43]: searching for similar users only in the data of target context. Hence recommendations can only be provided for users, who already expressed their preferences for predefined contexts, matching a target context either exactly or in a generalised form [43]. Therefore developing better understanding of how to use the context in the recommender systems is an important but largely unsolved problem [43].

The adaptation to the social context is considered more challenging than to the individuals, especially if the groups include people with significantly different personal preferences [44, 45]. Many researchers aim at utilising in the social context knowledge, acquired for a “being alone” context [20, 44, 46–49], whereas others develop methods to provide for heterogeneity between group members by predefining its typical degrees [16, 17] or via negotiations [50]. Also, it was suggested to exploit user preferences, acquired for one domain or task, for enhancing adaptation to a new task: for example, to use preferences for books in a movie recommender [51]. Therefore in this research area methods to transfer knowledge between contexts were proposed. As a group is more than the sum of its members [48], methods for using identities of group members as features [52] and methods to explore personality traits as features [53] were also explored in this area.

The recommenders can acquire training data explicitly, by asking the users to rank items, for example, movies, or their attributes, for example, genres and actors. The context, for which these preferences are acquired, is also often obtained explicitly or via sensors. Some systems instead acquire implicit user feedback by observing how the users deal with the recommended items: for example, select or skip them and view/listen fully or partially [54–56].

**Affective computing** aims at recognising human emotions, usually, by trained classifiers. Recognition results can be employed in* human-computer interaction, multimedia analysis*, and so forth. Due to the difficulty to understand human behaviour, data in this domain are usually labelled explicitly.

The affect recognition has to be adapted to personal differences and social rules because relations between communicating persons and customary ways to behave in different settings vary significantly. For example, in one context upset persons may scream and grimace, whereas in another context they may stay silent due to etiquette, the emotions having to be recognised only from facial expression [57]. Due to the difficulties to collect and annotate contextual data, however, the majority of human affect analysers remain context insensitive, as the recent surveys state [23, 58]. Most often, they are trained with data, acquired in very limited sets of contexts, and do not generalise well to other contexts. For example, capabilities of an audio-based emotion classifier to recognise several emotional categories (e.g., joy and anger) and distinguish between positive and negative arousal and valence were compared in [59] on six databases of spontaneous, induced, or acted emotions, collected in different countries. This evaluation has shown that the “*performance is decreased dramatically when operating cross-corpora-wise,*” mainly due to differences in displaying spontaneous and acted emotions in different contexts. Therefore this area offers methods of runtime training of context-specific models and methods of knowledge transfer between contexts. Adaptation to historical factors is also studied fairly often, mainly, to previous emotional states, being strongly interdependent with the current one (e.g., an excited person does not calm down instantly).

**User interaction (UI)** is concerned with providing convenient application interfaces. According to a recent survey [60], adaptation to personal preferences is an important future research direction. Adaptation to various definable and indefinable task factors (such as standing versus walking and answering a call versus a text message); social factors (e.g., in public speech interaction may be undesirable); and environmental factors (e.g., light) is also important [61]. Currently, the UI is mainly adapted to predefined computational contexts, such as screen size and device capability to deliver information via certain modality, for example, audio or video. Adaptation is most often based on rules, created by the application designers or end users: the former ignore the personal differences and the latter require the user efforts. Hence a recent review [60] suggested that interaction adaptation requires fundamental improvements, based on machine learning techniques. Nevertheless this area offers a few studies into knowledge transfer between contexts. Training data is acquired either implicitly via tracking customisation choices or explicitly by asking users to rank options or to perform certain tasks.

## 4. Basic Approaches and Examples of the Lightweight Adaptation

The lightweight adaptation is an emerging research area with a handful of approaches proposed to date. The lightweight adaptation is most beneficial for the application domains where (1) variety of users and contexts is large and (2) reasoning errors cause no serious problems for the users. This is usually the case with multimedia retrieval and recommender systems. Some additional insights into this problem can be found also in studies into multimodal fusion in other domains, for example, biometrics, and will be presented in this review, too. Although the context influence on human and algorithm behaviour is of the main concern of this paper, the adaptation to differences in users' personalities will be presented, too. For example, affect recognition should take into account that humans usually express emotions freer in informal than in formal settings. However, a reserved person always expresses emotions more subtly than an open one. In this case the adaptations to personal differences and “formal versus informal” context have no conceptual differences.

Some common approaches to decrease the user efforts, such as popular unsupervised and semisupervised learning methods, will not be surveyed here because they either require excessive computations or use too inflexible modelling assumptions. For example, semisupervised learning is often based on the assumption that points, located one near another in the feature space, belong to the same class [26]. However, it is well known that points, which are close to each other in one context, may appear quite distant in another context. This is why modification of similarity measure is a fairly common way to adapt to users and contexts [62, 63]. Furthermore, experimental comparison of conventional semisupervised learning with more lightweight context adaptation demonstrated that the latter can be significantly more accurate [37]. Unsupervised learning favours typical (statistically significant) data patterns and thus may fail to catch atypical context-dependent behaviours. Active learning methods are not surveyed, too, because they choose data samples, most informative for classification, but not necessarily easy for humans to annotate. Moreover, the perception of labelling difficulty depends on a person [29] and thus for end users it is more convenient to choose themselves which samples to annotate.

### 4.1. Classification of Adaptation Approaches

Context adaptation can be considered a generic machine learning problem of designing and training systems to perform well enough on data, acquired at runtime. However, while conventional learning assumes the similar enough runtime and training data, the situational adaptation may also require accounting for significant differences between the contexts. In designing a conventional system, one decides first whether to use a single classifier or an ensemble of multiple classifiers [64]. In the latter case, additional decisions are to be taken at the four design levels:

- Combination level: how to deal with outputs of the base classifiers (members of the ensemble), in particular how to select these members for each case and/or how to combine their outputs.
- Classifier level: which base classifiers to choose, for example, whether to employ same or different algorithms.
- Feature level: whether the same or different input features should be utilised by all the base classifiers and which features should be chosen.
- Data level: whether the same or different datasets should be used to train all the base classifiers and, if needed, how to choose the training data to select the best classifiers and/or optimise their combination.

Context-adaptive systems can use a single classifier or multiple classifiers, too, but additional decisions on using contextual parameters are needed in both cases. The context data in a single classifier can serve as an input feature or a latent variable. A multiple classifier system offers more choices: for example, either a single context-specific model can be trained for each context or multiple models can be trained for each context. Some or all the classifiers can also use the context as a feature or latent variable. These choices are very important because they determine the adaptation type: a multiple classifier system, training its own model for each context, can switch arbitrarily and abruptly between the contexts, whereas a single classifier system, using context parameters as features, can react more smoothly at context changes. Due to the need to reduce user efforts, it is also important to decide whether the data or models of initial contexts can be reused or only the target context data should be used for adaptation to this context: reusing data or models of initial contexts decreases the need in the target context data but may hinder the adaptation if the initial and target contexts differ significantly.

One of the proposed ways to classify adaptation approaches is to consider a number of employed inference models: adaptation in a multimodel system is a procedure of switching between models, whereas adaptation in a monomodel system is a procedure of tuning its parameters [65]. Another way [7] is to consider use of contextual data: context can be used as constraints (forbidden operations, probabilistic conditioning, etc.) or as additional features, semantics, or situation elements (e.g., context may change a meaning of information or bring new dimensionality into a problem). The work [65] does not list adaptation via selecting multiple models and combining their results, though, and the work [7] is concerned with use of context features rather than high-level situations. In addition, when a totally new situation emerges, it may be needed to train a new model from scratch.

**Figure 2:**
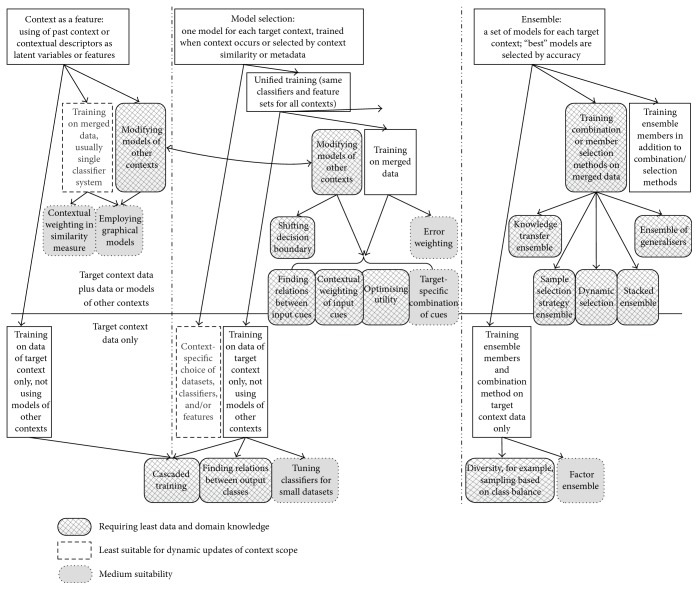
Lightweight adaptation approaches.

We suggest classifying situational adaptation approaches based on the choices on combination, data, and feature level, and we suggest three major groups, called in brief* model selection*,* ensembles*, and* context as a feature*.* Model selection* group encompasses procedures of using contextual data for selecting a single model from existing models, as well as methods to train a new model from scratch.* Ensembles* group encompasses procedures of selecting one or several models from existing models based on their accuracy rather than contextual data, as well as methods to combine their results.* Context as a feature* group encompasses methods to use contextual data as input features (additional dimensions) or latent/hidden variables.  Figure 2 presents most interesting approaches from these groups along with our understanding of their ability to handle situations, emerging at runtime. The following sections give more details. Other choices on the classifier and feature levels are influenced by the aforementioned choices and by peculiarities of a problem at hand in ways similar to the conventional systems. The majority of the lightweight adaptation methods presume the same feature sets in all the contexts, but some approaches allow for employing the same feature extraction methods in all the contexts even if this results in context-specific sets of cues.

### 4.2. Model Selection

The* model selection *group embraces multiple classifier systems, where only one model is trained for each context. Metadata or contextual values, describing the context each model was trained for, can be stored along with the models to retrieve them by metadata or context similarity. The contexts can be recognised from sensor data or user interaction (the latter gives only a coarse situational description). If the context cannot be recognised or is unlikely to emerge again, the old models can be discarded. The models can also be built for overlapping or nested contexts to either retrieve the best match or combine several suitable models. Depending on the data usage, this group can be further categorised as follows:

- *Context-Specific Classifiers and Data*. Each model is trained on the target context data and context-specific features and/or reasoning methods are employed. Lazy methods search for similar cases only within the target context, using context-specific distance measures.
- *Context-Specific Models and Data*. Each model is trained on the target context data, but feature selection and reasoning methods are the same for all contexts. Lazy methods search for similar cases only within an appropriate data part, but the distance measure is the same for all contexts. This category may be less adaptive than the previous one, but it is more lightweight because although the models have to be retrained from scratch for each new situation, no additional designer efforts and data are required for the feature and/or algorithm selection.
- *Context-Specific Models with Mixed Data, That Is, Knowledge Transfer*. Feature selection and reasoning methods are same for all contexts, and knowledge of other contexts is used to build models for the target context; for example, models can be trained on the merged data. More sophisticated methods of knowledge transfer on data, feature, combination, and model levels were also proposed, mainly in research areas called “transfer learning” and “domain adaptation” (in these areas the terms “domain” and “task” are typically used instead of “context”). Data- and feature-level knowledge transfer methods are usually computationally expensive (see, e.g., [5, 37, 66]). Knowledge transfer on combination level is usually done by fusing together outputs of the models, trained for different contexts, for example, with weights depending on context similarities. To obtain a target context model by the model-level knowledge transfer, selected parameters of models for initial contexts are modified. Lazy methods search for similar cases within the data from different contexts and treat samples from these contexts in a different way. Knowledge transfer saves data collection efforts significantly, and the model-level one further reduces data collection needs and training time because usually not all the parameters of the initial models are modified.

While the first two of these approaches suit both similar and dissimilar contexts, the knowledge transfer suitability to dissimilar contexts depends on the transfer type and degree of preserving the old knowledge, for example, on constraints on modified model parameters.

#### 4.2.1. Context-Specific Models and Data

(1)* Contextual weighting* employs *n* generic cues, or individual data modalities, with outputs *S*_*i*_, and combines them linearly, ∑_*i*=1_^*n*^ *w*_*i*_ *S*_*i*_, with context-specific weights *w*_*i*_. This approach is often used in audio-visual data analysis [11, 25] and fusion of data from multiple sensors [7]. For predefined situations the weights can be determined from the prior knowledge; for example, the video modality can be assigned higher weight than the audio modality for daytime and lower at night. The weights can be also calculated using quantitative estimates of the accuracy *a*_*i*_ of each modality in the target context: *w*_*i*_ = *a*_*i*_/∑_*j*=1_^*n*^ *a*_*j*_.

Alternatively, for adapting to changes in reliabilities of modalities without relying on domain knowledge and training data, the weights can be estimated from stream entropy [25] or relations between outputs of the different modalities, for example, the variances of several topmost scores [25], using rules of the kind “if the topmost and the second best scores differ less than some threshold, then the weight *w*_*i*_ is high… else….” A fairly small training dataset can suffice for either the accuracy estimation or the rules' derivation.

The lightweight adaptation to a user query in the multimedia retrieval tasks is performed via either “feature relevance estimation,” that is, modifying the feature weights, *w*_*i*_, in a linear similarity measure, or “query vector modification,” that is, adjusting the feature weights in a query vector [36]. The weights can be adapted heuristically or by genetic algorithms; the latter require training data, but no domain knowledge. Cues can be combined also in other ways than a simple weighted sum. For example, Calumby et al. [67] employed genetic search for the best combination of various text-, colour-, and texture-based features and their functions including, in particular, products and square roots. This search aimed at minimising the classification error for the labelled items, and the training data consisted of 55 images. A few dozens of user-labelled items are fairly typical database size in multimedia retrieval as users do not provide abundant training data. Therefore contextual weighting is a fairly common approach because, for example, probabilistic approaches, such as Bayesian inference, require more feedback data [68].

(2)* Optimising utility function* is achieved by selecting from a large set of generic cues a subset with the highest utility. Utilities of each subset depend on the target context and can be easily computed. Usually, the utility functions are application-specific sums of context-dependent (often, heuristic) values *S*_*i*_, reflecting gains or losses of including different cues and/or their combinations in the subset. Adaptation is based on calculating the utilities of various combinations ∑_*i*∈*C*_ *S*_*i*_ and optimising (possibly with constraints) the result to choose the subset *C*_opt_. This approach does not require training data. For example, for GUI (graphical user interface) adaptation task values *S*_*i*_ may reflect costs of satisfying/ignoring user preferences, easiness of navigating between GUI elements, and so forth, and the optimisation may result in selecting, for example, a picture box and a small text box with a scroll bar in one context and selecting a large text box with no scroll bar in another context.

This approach was employed for user interface adaptation [69–71]. In [69] GUI was adapted to different screen sizes and to user preferences and abilities, for example, special needs of motor-impaired users. Costs and constraints of choosing different elements were estimated by tracing performance of each user, for example, speed of clicking on interface elements of different sizes. However, the constraints elicitation required fairly long and diverse interaction histories: in the tests able-bodied and motor-impaired participants had to perform tasks during at least 25 and 30–90 minutes, respectively [72]. The interfaces were adapted only to fairly similar task contexts, like controlling light intensity, ventilator, and audio-visual equipment in a classroom, and only for an individual GUI usage.

A greater variety of contexts and interaction modalities were considered in [70]: contexts included environmental, task, and computational factors, such as light, weather, noise, motion, screen size, and keyboard type, and I/O modalities included eye tracking, gestures, audio, video, and vibration. Explicit user preferences were acquired by asking the users to manually assign numerical scores for different interface elements in various contexts. This process is not very easy for the users, however, and more error-prone than, for example, selecting the most appropriate elements from available options. Hence in the work in interface adaptation to different platforms [71], utilities of different GUI options (e.g., different font sizes) were partially obtained from users and partially specified by system designers.

(3)* Tuning classifiers for small datasets*: special efforts are taken for selecting data features, classifier parameters, or training samples to reduce negative effect of small data size. This approach was proposed for multimedia retrieval with SVM (support vector machine), and tuning was performed by selecting SVM kernel or subset of training items [63, 73]. SVM training was done for each query in a standard way, by minimising the classification error for the user-labelled items. In particular, an iterative user's feedback in [73] required every user to label nine retrieved images per iteration and achieved a reasonable precision after 6–8 iterations. Video retrieval in [63] gave the users 15 minutes per query for evaluating and labelling the obtained query results.

(4)* Cascaded training* uses first unlabelled data for initial parameter estimates and then the labelled data for fine-tuning. This approach was applied to deep neural network [74], discrete HMM (hidden Markov model) [75], and MLP (multilayer perceptron) [26] based classifiers. The work [74] mainly aimed at increasing accuracy of offline training; difficulties to obtain the labelled data were not of main concern. The work [26] reviewed approaches for reducing the need in labelled data, but also mainly for offline training. The work [75] was concerned with user-controlled runtime adaptation to indefinable situations (social behaviours) and therefore aimed at finding a quick and lightweight adaptation method. The main goal was to detect show highlights by recognising arousal of a show audience. Classification was performed by HMM, employing the cues from audio-visual analysis (such as laughter, speech, silence, noise, and human motion) as observations. The proposed classifier needed to distinguish between three arousal levels in fairly dissimilar contexts: show types with notably different ways to express excitement (a concert, a circus, and a sport match). Furthermore, the data were collected in uncontrolled settings, and the classifier had to deal with considerably different audio-visual backgrounds and missing behavioural cues. Hence a new model was trained each time after a new context emerged and the user provided annotated examples.

During the first stage an HMM model was obtained for each context in conventional way, by using Baum-Welch algorithm. During the second stage of cascaded training only the observational probabilities of the HMM were optimised with a differential evolutionary algorithm, aiming at minimising the classification error for the labelled data (see also Section 4.2.2). The HMM employed the maximum posterior marginal (MPM) decisions, rather than the conventional maximum* a posteriori* (MAP) ones, to achieve more robust adaptation. As a result, the adaptation did not require significant time and efforts: the annotations were collected for 10 minutes per context, and the annotator was free in choosing samples to label. In the tests as little as 25 labelled samples per context (5–12 samples per class in each context) allowed significantly increasing the classification accuracy, comparing with the conventional HMM training. Comparing with an alternative full-scale adaptation, this fusion-level-only one relieves the users of having to make considerable efforts for recognising and labelling selected behavioural cues in the data, containing large number of mixed sounds and video backgrounds.

(5)* Learning context-specific relations between the outputs of a multiclass classifier* [76] is proposed for multimedia analysis. Because the concept detection is a multiclass classification with nonexclusive classes, its accuracy can be increased by learning the most frequent cooccurrences of classes in different contexts. In the hierarchical system in [76] context-independent concept (class) detection models are trained first. Then an affinity graph with a node for each concept is built to learn context-dependent relations between the concepts. Each two correlated nodes form an edge, the six strongest edges for each node being kept. How many training samples are needed to learn such a graph was not mentioned in [76], but as a whole, the context-specific learning was very fast: less than a minute comparing to tens or hundreds of hours, required usually to completely retrain the concept detection models.

#### 4.2.2. Model-Level Knowledge Transfer

A lightweight model-level knowledge transfer for trained classifiers is most often done by shifting a decision boundary. Transfer design requires making three major choices: (1) which parameters of initial models are modified (all or just certain selected parameters); (2) how parameters are modified (choice of an objective function and an algorithm to optimise it); and (3) which initial models are modified (e.g., a model trained on the merged data of all previous contexts or a model of a certain context).

(*1) Optimising model parameters:* two fairly generic ways to shift decision boundary of trained models have been proposed to date: evolutionary algorithms and gradient descent based search for changes in model parameters, minimising classification error in target context. Most often, in this approach only training data for the target context is employed. To increase accuracy of interactive image segmentation, evolutionary optimisation of segmentation parameters in [77] was based on the user's feedback on whether too many or few image segments were obtained. At each iteration the users were presented with a small number of images, for example, six images, and on average 7–10 steps allowed to achieve satisfactory segmentation accuracy.

The evolutionary optimisation was used also in [75] for modifying a discrete HMM based on the MPM decisions. This work studied two approaches to adapt an affect recognition system: cascaded training (see Section 4.2.1) and model-level knowledge transfer. In cascaded training initial models for each target context were trained in conventional unsupervised way by Baum-Welch algorithm. In model-level knowledge transfer a model, trained on data of some other context, served as initial model for the target context (the contexts are described in Section 4.2.1). In both approaches the initial models were modified by evolutionary algorithm to increase accuracy of recognising arousal levels. Let the goal arousal levels be associated with *K* hidden states {*θ*_*k*_:  *k* = 1,…, *K*} and let **X** denote a space of the observed vectors **x** of cues for each state *θ*_*k*_. Given observational probabilities **p**_obs_ = [*p*(**x**∣*θ*_*k*_; **α**_*k*_):  *k* = 1,…, *K*; **x** ∈ **X**] with the unknown parameters **A** = {**α**_*k*_:  *k* = 1,…, *K*}, the initial training was used to estimate the *K* ^2^ interstate transitional probabilities **p**_tr_ = [*p*(*θ*_*k*_∣*θ*_*l*_):  *k*, *l* = 1,…, *K*] and parameters, **A**, of the observational probabilities for the HMM. Then the evolutionary algorithm modified only the observational probabilities to minimise the number of classification errors on the user-labelled data for the target context. In the tests as little as 25 labelled samples per each target context considerably increased the classification accuracy in comparison with the nonadaptive or context-independent models, despite a fairly significant difference between initial and target contexts.

Unlike the work [75], Caridakis et al. [78] studied emotion recognition of individuals, and in their data contexts did not significantly differ from each other: all the data consisted of records of persons, communicating with four artificial computer characters [79]. The emotional expressions were not very intense because all the records were acquired in the same laboratory and communications with the artificial characters do not follow exactly the same social rules as with real humans [79]. The records were classified into *K* classes of emotions by a neural network (NN). Its parameters, **w**, had been learnt initially in a fully supervised mode using a special training set of the labelled records. For each input vector** x** of cues, the NN forms the *K*-component output vector **f**_**w**_(**x**) = [*p*(*k*∣**x**):  *k* = 1,…, *K*; ∑_*k*=1_^*K*^ *p*(*k*∣**x**) = 1] of class probabilities. To adapt to a new target context, the initial parameters are changed incrementally by minimising, with the gradient descent search, a weighted total error *ε* = *ε*_target_ + *λε*_ini_ on the initial (**x**_ini:*t*_) and target (**x**_*t*_) labelled inputs, the weight *λ* determining significance of the target context data for adapting the NN:

(1) εtarget=12∑t=1Ntargetfwxt−ptarget:t,εini=12∑t=1Ninifwxini:t−pini:t,

where **p**_target:*t*_ and **p**_ini:*t*_ are the desired output *K*-component probability vectors for the labelled inputs, |⋯| denotes the vector norm $\left|z\right|=\sqrt{z_{1}^{2}+\cdots +z_{K}^{2}}$, and *N*_ini_ and *N*_target_ are cardinalities of the available initial and current training datasets:

(2) Dini=xini:t,pini:t:  t=1,…,Nini,

(3) Dtarget=xt,pt:  t=1,…,Ntarget,

respectively.

The adaptation was lightweight because only small increments of the weights **w** were allowed, and nonlinear signal transformations in each neuron were linearized using first-order Taylor's series decomposition. Due to linearization, the increments of the NN parameters were obtained by solving a system of linear equations with coefficients depending on the initial parameters and all the training data in the datasets *D*_ini_ and *D*_target_. The latter one contained segments with a steady emotional state of the user during 50 or less video frames. Using the dataset *D*_ini_ for the initial contexts reduces the need in the labelled target context data *D*_target_ but might hinder the adaptation in the case of significant differences between the initial and target contexts.

(*2) Algorithm-specific methods to shift a decision boundary*: in these cases, already trained classifiers are modified by model-level knowledge transfer methods, specific to the algorithm, conventionally employed for training these types of models. For example, quadratic programming can be employed for modifying the SVM [37, 80, 81] and Expectation-Maximisation can be employed for modifying the HMM [82]. High-level visual concepts, related to TV news videos, were detected in [37, 80, 81] with the classifiers, based on the binary SVM:

(4) $$\begin{array}{c} k=\mathrm{sign}\left[\;f_{w;c}\left(\;x\;\right)=\overset{n}{\underset{i=1}{\sum }}w_{i}\varphi_{i}\left(\;x\;\right)+c\;\right]\in \left\{\;-1,1\;\right\}, \end{array}$$

where *f*_**w**;*c*_(**x**) is a decision boundary or a separating hyperplane with coefficients **w** and offset *c* in the *n*-dimensional space of kernel functions *φ*_*i*_(**x**) or features of the observed vectors of cues, **x**. Initially, the classifier was trained on a large labelled set *D*_ini_ = {(*k*_*t*_, **x**_*t*_):  *t* = 1,…, *N*_ini_} of *N*_ini_ data items from one TV domain. The quadratic programming based training determined the coefficients **w** = [*w*_1_,…, *w*_*n*_] of the hyperplane that separates the set *D*_ini_ with the largest margin and correctly classifies data points of both classes in the presence of a tolerable fraction *γ* of errors {*ε*_*t*_:  *t* = 1,…, *N*_ini_}:
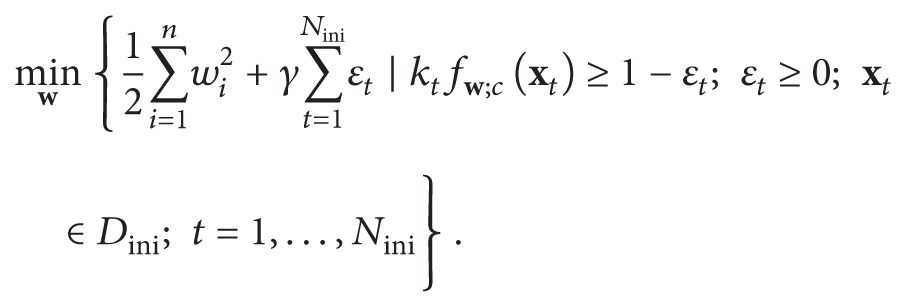
(5)Simultaneously, the initial training specified a subset *D*_sup_ of *N*_sup_ support vectors, located just at the margin distance at both sides of the separating plane.

The adaptation to the new domain in [81] was done by adding to the initial classifier *f* ^*∗*^(**x**), learned from *D*_ini_, a shift Δ_**w**;*c*_°(**x**) = ∑_*i*=1_^*n*^ *w*_*i*_ *φ*_*i*_(**x**). The latter was learned in [81] from the labelled new dataset *D*_target_ of the size *N*_target_ with due account of the obtained complete classifier *f*_**w**_(*x*) = *f* ^*∗*^(**x**) + Δ_**w**_°(**x**) and by using the same optimisation framework as the SVM:
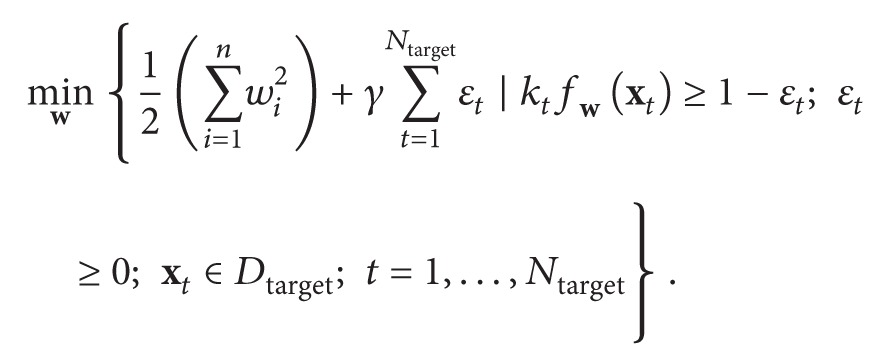
(6)This approach aims at placing the new decision boundary close to the initial boundary *f* ^*∗*^(**x**) = 0, probably, because all the news channels under consideration did not notably differ in this work. In the experiments in detecting 39 contexts only from one to ten explicitly labelled samples per concept were used, and the function-level knowledge transfer achieved nearly the same accuracy as the best amongst the more computationally expensive techniques for building context-specific models (more details are provided in Section 4.5).

In cases of notably different initial and target contexts placing a new decision boundary close to the initial one may hinder the adaptation. Hence in [80] a different SVM-specific adaptation approach was suggested: to retrain the models on the combined dataset *D*_com_ = *D*_target_ + *D*_sup_ of the size *N*_com_ = *N*_target_ + *N*_sup_. This dataset is much smaller than the initial one and contains both the labelled new dataset *D*_target_ of the size *N*_target_ and the set *D*_sup_ of the initial support vectors. An application-dependent measure *s*(**x**) of relative similarity of each support vector **x** from *D*_sup_ to the current dataset *D*_target_ was used to reduce the impact of classification errors for these vectors onto the updated coefficients **w**:
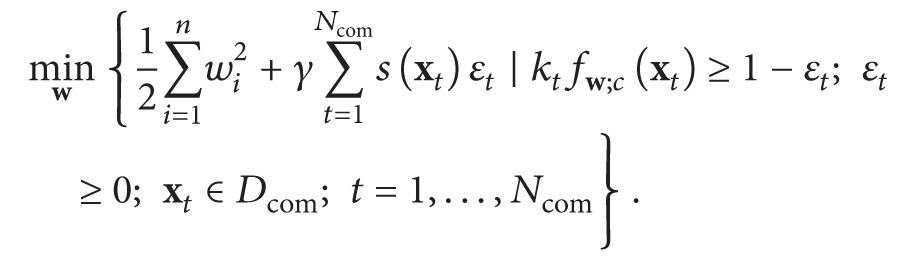
(7)The employed similarity measure decreases the influence of the support vectors, being located far from the current dataset, onto the tolerable errors:
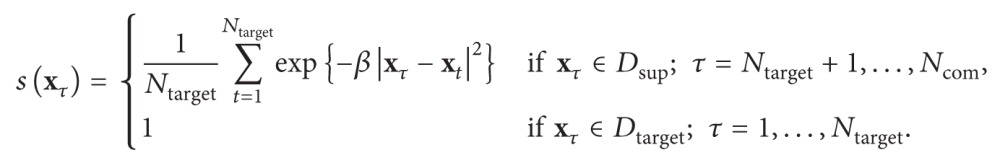
(8)The control parameter *β* ≥ 0 is selected empirically in order to obtain a reasonably high overall performance (Jiang et al. [80] suggested choosing *β* based on a series of systematic validation experiments). If *β* is small, all the vectors from *D*_com_ almost equally take part in the adaptation; that is, the SVM is simply retrained using all the new labelled data together with all the initial support vectors. Hence small values of  *β* better suit cases of fairly similar initial and target domains. The larger the *β*, the lesser the influence of those support vectors which are far from the new dataset *D*_target_. Therefore, choosing a small parameter for the dissimilar initial and target domains would hinder adaptation, while choice of larger values of *β* would decrease size of the most influential part of the dataset. To compensate for this decrease, it would be desirable to obtain additional data samples for the target domain.

Which of the available initial models should be adapted to the target context is an important but, due to required notable efforts, rarely explored problem. As an infrequent example, a few ways to select the most appropriate model to adapt are compared in [37, 81] with building a target context model from multiple initial models. The ways to select an initial model include (1) comparisons of data or score distributions; (2) classification accuracy comparisons of each initial model and its ensemble, which should be better, by usual assumption, than any of its members; (3) linear regression-based predictions of the initial models' accuracies in the target domain; and (4) iterative user feedback to evaluate how quickly different adapting models improve with the existing labelled data (a history of fast improvement can provide more labelled samples to the model at the next iteration). The multiple initial models appeared to be beneficial in the tests, but none of the tested schemes outperformed others, or even a randomly chosen adaptation significantly (probably, due to relatively small differences between the contexts).

To circumvent selecting the best candidate among the existing initial models, a so-called general model (i.e., a model trained using merged data for all initial contexts) can be adapted instead. That adapting such a general model is feasible has been confirmed for the HMMs in [75] (described in this section above and also in Section 4.5) and in [82]. In [82] the event-specific models for detection of different meeting events, such as note taking and discussion, were obtained from a general model via maximum likelihood estimates of parameters. These estimates required small amounts of the labelled target event data: less than a minute of audio-visual recording per event.

(3) Although some works employed modification of all model parameters for adaptation (e.g., [78, 81]),* adapting only selected parameters *under assumption that other parameters are shared across allcontexts is more common. Proper selection of parameters to adapt requires certain domain knowledge but notably reduces the need in training data. For example, in the model-level knowledge transfer of HMM often transitional probabilities for the hidden states are shared, while the observational ones are adapted: either these probabilities for a discrete HMM directly, as in recognising the show audience excitement [75], or related probabilistic parameters of continuous state-observation relations, like the emission parameters of the Gaussian mixtures in recognising the meeting events [82]. Feasibility of this approach was demonstrated not only for fairly similar initial and target contexts but also for fairly dissimilar ones [75]. In recommender systems* adapting only selected parameters* approach employed an assumption that generic user interests are valid in all contexts [83]. This approach was tested for two quite different initial and target contexts: the generic user interests were inferred first from TV viewing histories and then tourist attractions of the same types were recommended (e.g., diving, for the users watching TV programmes about water sports).

#### 4.2.3. Data-Level Knowledge Transfer

Unlike model-level knowledge transfer methods, transfer methods on data level require training of a new model from scratch: their main goal is to reduce data acquisition efforts.

(*1) Error weighting*: training the models on a merged dataset, but weighing classification errors on the initial and target contexts differently. This approach suits quite similar contexts and requires either domain knowledge or additional data for defining the weights: too small weights of the old knowledge nearly ignore it, while too high ones hinder proper adaptation. The NN-based affect recogniser in [78] (described in Section 4.2.2) adapted model parameters by minimising a weighted sum of errors on the old and new data. An SVM-based multimedia analysis system [80] (described in Section 4.2.2) was trained on a dataset, containing both labelled target-domain data and support vectors of the initial model, weighed according to their distances from the new training samples (the larger distances are penalised).

(*2) The use of model parameters as training data*: the learned parameters of an initial model are added to the training data in an algorithm-specific way. In particular, the above SVM-based system [80] for multimedia analysis is adapted by training on the dataset, containing the target-domain data and the support vectors of the initial model.

#### 4.2.4. Lazy Classifiers

For nontrained classifiers the following knowledge transfer approaches were proposed.

(*1) Vector modification*: many systems store user preferences in a form of vectors, where each element denotes influence of a certain modality on a final result, for example, importance of a certain image feature for the current query and degree of user liking/disliking of a certain interaction modality, movie genre, and so forth. Prediction of a target context vector **S**_*i*_^target^ can be done by shifting vector **S**_*i*_^ini^, obtained for some initial context: **S**_*i*_^target^ = **S**_*i*_^ini^ + Δ_*i*_, where the shift Δ_*i*_ of the initial preference vector can be the average difference between the preferences of other users for the two contexts [47], or the classification error minimiser found by differential evolutionary optimisation [84]. Usually, a preference vector of whatever available initial context is modified, and all its elements are modified. This approach requires training data of both initial and target contexts, but fairly little data may suffice: preferences of 20 users served as training data in [84] and preferences of 33 users in [47]. In spite of its simplicity, this approach was successful in predicting user preferences for predefined contexts regarding interface modalities [84] and regarding tourist attractions [47]. In the latter work, variety of usage contexts was fairly large, for example, weather, budget, travel goal, and travel companion.

(*2) Modifying a similarity measure*: to increase the classification accuracy, the most common linear measures, ∑_*i*=1_^*n*^ *w*_*i*_ **x**_*i*_, are adapted to different contexts by heuristic or evolutionary optimisation of the weights, *w*_*i*_, of cues, modalities, or samples, **x**_*i*_, observed for these contexts. Heuristics is usually based on domain knowledge, while use of evolutionary algorithms usually requires both the initial and the target context data but allows dealing with both similar and dissimilar contexts without estimating the context similarities. Assigning different weights to the same observations for different contexts relaxes the similarity assumption of the CF but provides for no case when this assumption completely fails due to significant differences between initial and target contexts.

To adapt to nondefinable computational factors (different databases of movie ratings) in CF-based recommenders, the work [51] proposed several ways to account for correlations between the contexts in the user similarity measure. Adaptation to nondefinable computational factors (different databases of movie ratings) with optimisation algorithms was successfully employed in [62]. This result demonstrated that* modifying a similarity measure* by an optimisation algorithm does not require notable efforts from each user: in recommender systems the users provide as many ratings as they want, but one user rarely provides many ratings. Adaptation to both definable and nondefinable contexts (individual and group interaction with two fairly different applications: cooking and car servicing assistance) with optimisation algorithms was successfully employed also in [84], where very little training data was used: interaction preferences of 20 users only.

(*3) Target context-specific combinations of cues, obtained in other contexts*: the cues, obtained for one or more initial contexts, can be combined in different ways, being specific to the target context: the weighted average, voting, rule-based heuristics, and so forth. One rather simple combination scheme for the recommenders, called “post-filtering” [43], uses context-independent recommendation methods, just as prefiltering. But unlike prefiltering, postfiltering gives recommendations on the basis of data, acquired in all contexts, and then either filters or reranks these recommendations for the target context. For example, music recommendations can be provided using all available data and then reranked in line with a historical context, namely, the last songs just listened by the user [85]. Which technique to choose depends on a system goal, as comparison of the prefiltering and postfiltering techniques did not result in a clear winner with respect to different evaluation metrics [86].

Adaptation to social context (group use of an application) often utilises user preferences (cues) obtained in context of individual use of an application. Individual preferences of group members can be combined in various ways [20, 44, 45, 48, 49], but these ways often fail to adapt to heterogeneous groups. A data-adaptive way is to learn, for example, with an evolutionary optimisation, to what extent different input cues may influence the classification in various contexts to find the dominant cues. For example, influences of the group members on group ratings can be learned with a genetic algorithm, using data collected during group and subgroups sessions [87]. However, because collecting the subgroups ratings takes rather long time, this learning was tested on simulated data only [87]. To avoid data collection, designer-defined rules can be used for estimating social dominance and influence of each family member on group ratings, for example, based on age, social role (father, mother, etc.), and income [88]. But due to individual differences and cultural diversity the universal (i.e., suitable for any family) rules are difficult to define.

All the above model-level knowledge transfer scenarios, for example, adapting only selected parameters of the models, can be combined with other model modification methods and various methods to build effective training datasets.

### 4.3. Ensembles

Lightweight adaptation of ensembles can be performed by selecting the most appropriate base classifier(s) for each context and/or ways to combine their outputs. Context need not be recognised in this approach: ways to select or combine base classifiers depend on their performances in the target context. The ensembles differ basically in two aspects:

1. Whether outputs of all base classifiers are combined or the best classifier is selected and also, in the latter case, how the base classifiers are selected for each test sample, say, by accuracies on all training data or achieved only for similar samples.
2. What methods are used to update the ensemble at runtime, for example, only the classifier selection and/or combination rules and/or parameters of the base classifiers can be adapted; and/or some new classifiers can be added, while the underperforming ones can be removed.

Selecting the classifier is the most lightweight option, but it does not accommodate new knowledge easily, whereas updating the base classifiers corrupts the old knowledge. Updating classifier pools keeps both the old and the new knowledge, but optimisation of such pools requires additional training data. Naturally, all these approaches can be combined.

The ensembles differ also by the data usage:

- *Context-specific ensembles* train and evaluate their members and selection/combination methods on the target context data. Lazy members search for similar cases only within the target context. This approach suits both similar and dissimilar contexts. Such ensemble may require more data or time to train and test the base classifiers compared to training* context-specific models *(described in Section 4.2.1) but ensures the more accurate adaptation if the base classifiers are diverse enough.
- *Mixed data ensembles* train their members and/or selection/combination methods on both the initial and the target context data and evaluate them on the target context. Lazy members may search within the different contexts or employ different search techniques. Such ensembles significantly reduce the data collection efforts, but their ability to handle both similar and dissimilar contexts depends on specific capabilities of the ensemble members.

Context-specific ensembles usually employ combination of the members' outputs. In the mixed data ensembles, if different members are trained on the data of different contexts, such combinations may work for sufficiently similar contexts. For example, recommender systems often deal with a concept drift (change of the users' interests over time) this way, but then the base classifiers are usually completely retrained at runtime [89, 90]. However, in the mixed data ensembles classifier selection is more appropriate than classifier combination because in the former case the ensemble does not fail if just one member suits well the target context, whereas in the latter case the ensemble fails if the majority of the members fail. The selection of the best member is a good option in the context-specific ensembles, too, because it can be more accurate than classifier combination if trained well [64].

#### 4.3.1. Combination-Based Ensembles Using Context-Specific Data

(1)* Diversity-based ensembles*: training base classifiers on different data chunks or employing different classification algorithms. This is a well-known way to increase the accuracy in conventional ensembles. This approach was used in a multimedia retrieval system [40] to adapt to different user queries: an ensemble of the SVMs was trained on the target context data in such a way that each SVM was trained on all labelled positive examples and negative examples, randomly selected from the unlabelled data (so that different training datasets include different negative examples). Then the ensemble was used to remove wrongly labelled samples: each SVM classified all positive examples, and those out of them, classified negatively by all the SVMs, were considered wrongly labelled. Then a new ensemble was trained on the reduced training dataset, and the final classification combined the outputs of all the SVMs because the ensembles are generally robust to noise. In the tests only five positive training samples for each query were needed, and the training times were short [40]. But the latter times may notably increase for the larger training sets.

This approach was employed also in TV recommender system in [52] to account for differences in viewing habits of different families. Ensemble contained SVM and CBR (case-based reasoning) classifiers, and each classifier employed as features contextual parameters: time and IDs of family members, watching TV. In the tests on 5 months real-life viewing data of 20 families ensemble achieved higher accuracy than any of its members due to their diversity: SVM was less sensitive to nondiscriminative input cues (e.g., in some families choice of programmes depended on time more notably than on presence of family members), whereas CBR better adapted to peculiarities (e.g., cases when choices of {A, B, C} subset of family members notably differed from choices of {A, B}, {B, C}, and {A, C} subsets). This result shows that accuracy of context adaptation may be increased by employing base classifiers with different degrees of sensitivity to nondiscriminative input cues and capable of building decision boundary both locally (as CBR does) and globally (as SVM).

(2) In the* factor ensembles* each base classifier models a certain context-independent factor affecting the final classification result, the factors' weights (in the weighted average, voting, or other fusion methods) being adapted to the context in order to increase the accuracy on the target context data [52, 55, 91]. The training datasets can be quite small if the number of these factors is small. For example, the retrieval of sport videos in [91] used a combination of several generic attention models, such as those based on camera motion and object detection. The models' weights were adapted to each user based on his/her feedback. The recommenders can also employ this approach; for example, the base classifiers for TV recommenders can be trained on different programme attributes (a genre, a channel, etc.) [52, 55], and the combination of their outputs can be adapted using the feedback data.

#### 4.3.2. Combination-Based Ensembles Using Mixed Data

Mixed data ensembles have been employed for adaptation to personal differences in expressing pain. Chen et al. [92] suggested a method to* optimise a pool of classifiers*,* trained on data for different contexts*: first, for each person in a training dataset a separate ensemble is trained with Ada-Boost algorithm to optimise pain recognition rate of this person. All resulting base classifiers of all subjects then constitute a classifier pool, and adaptation to a new person is performed by optimising weights of classifiers in this pool, also by AdaBoost. Optimisation aims at minimising error rate for the target person and is very quick because new base classifiers are not trained at this stage (only existing ones are combined). In the tests the proposed ensemble adaptation took 0.16 minutes per subject on average, and fairly small number of labelled samples per target person (from 25 to 50 samples) allowed achieving notably higher accuracy than that of a generic model. Unfortunately the paper does not explain whether ways to express pain differed significantly between the test subjects and how the proposed method dealt with the persons, most different from the others.

#### 4.3.3. Selection-Based Ensembles

(1) Base classifiers in an* ensemble of generalisers* are trained on the datasets that generalise the target context in different ways, for example, from a nearly exact match up to a mixture of all contexts, and lazy classifiers employ different prefiltering ways to generalise the context [46]. None of these cases requires large datasets for the target context. Success of handling of significantly different contexts depends on the database coverage. The CF-based recommender in [46] included both a general context-independent user profile and content-dependent ones, created using different schemes of generalising the context: for example, for predicting user preferences for a particular “Tuesday” context ranks, acquired on other workdays, were also used. Tests demonstrated the feasibility of including the general profile in such ensembles: it was selected in more than 50% of cases, due to either too weak dependences of the user preferences on some contexts or too small training datasets for these contexts.

(2) Unlike the majority of other ensemble types, employing pattern recognition methods as base classifiers,* knowledge transfer ensembles* employ as base classifiers different strategies to transfer knowledge between the initial and target contexts, for example, mappings between their input cues, or mappings between their output classification results, or mappings between their feedback utilisation models. Such ensembles require both initial and target context data. An ensemble of three relevance feedback techniques for image retrieval in [68, 93] adapted to each query by selecting the most appropriate technique or a combination of the two top-ranked techniques via either reinforcement learning [68] or particle swarm optimisation (PSO) [93]. The training data for each image retrieval query is never large.

A knowledge transfer ensemble in [31] predicted user interface preferences for new applications, screen sizes, newly assembled user groups, and so forth. Each ensemble member modelled a certain human strategy for a new situation: for example, to behave in a target context just as in the initial one; or as the majority of other users in the target situation irrespectively to the initial one; or as those among other users, which behaved similarly in the initial context. The first strategy suits similar initial and target contexts; the second strategy suits the target contexts, strongly affecting the users' preferences; and the third strategy transfers users' similarity. The pool of group strategies included also a few other behaviours, for example, the majority voting. The prediction was based on the known preferences of (1) a target user or group for a single initial context and (2) from 9 to 43 other users or groups for the same initial and target contexts. In the tests with three different applications (a cooking assistant, a car servicing assistant, and a recipe recommender) the interface preferences for each application were predicted based on knowledge regarding either very similar initial context or very different one. For example, preferences for interacting with the recipe recommender were predicted based on the known preferences for significantly different interface of the car servicing assistant.

Which transfer strategy best suits the current context transition and each interface element was estimated from data of user communities. For example, early technology adopters may use a broad range of interaction modalities in many applications, whereas similar attitudes towards healthy lifestyle may lead to similar preferences regarding wellness tips in shopping and cooking aids. However, similar attitudes towards technology adoption do not necessarily imply similar attitudes towards healthy lifestyle. When user similarity is preserved across the contexts and when other strategies are more appropriate follow from the user community data. Required amounts of training data depend on the ensemble size. No domain knowledge is required, provided that users in the database have similar culture: otherwise, their behaviour in new contexts could be too inconsistent to rely on the best ensemble member. This approach may fail also if ensemble members do not cover a sufficient range of the knowledge transfer strategies. On the other hand, it allows adapting to any new situation if different users use similar tags for similar situations, and predefining primary data types is not required provided that some ensemble members can handle new data types. In the tests the ensemble successfully handled transitions between similar and different initial and target contexts and outperformed each of its members.

(3) Base classifiers of the* sample-selecting ensembles* are not pattern recognition methods either: they model different strategies of choosing training samples, for example, between the classes or between the samples of various contexts. Such ensembles may be context-specific or use mixed data and may handle both similar and dissimilar contexts. For example, the aforementioned model-level knowledge transfer in [37, 81] allows for only small shifts of the SVM decision boundary (described in Section 4.2.2). This constraint may hinder the adaptation if the initial and target contexts have significant differences. An alternative approach to SVM adaptation for video concept detection in [94] is to train a model on a dataset, containing the labelled samples from both the source and the target domains, and to select from two strategies of choosing training samples: either near to the decision boundary of the initial model or from most unlikely ones to come from the initial domain data distribution (found by conventional kernel density estimation). The former and latter strategies better suit similar and dissimilar initial and target domains, respectively. To avoid the domain similarity estimation, the samples in [94] were selected as follows: if more samples of a certain type got positive labels at current iteration, a larger proportion of the samples of this type was chosen at the next iteration. The required amounts of the training data depend on task complexity. This approach outperformed the approaches of [37, 80, 81] (described in Section 4.2.2) in experiments on detecting 36 concepts in two rather different video collections. But the adaptation was not very quick, as it required ten iterations.

Originally, the ensembles of sample-selecting strategies were proposed for active learning, that is, for selecting samples from unlabelled data. As it is easier for the users to choose by themselves the samples to annotate, an obvious modification, which can be suggested, is to present more samples than necessary and allow the users to choose and annotate only a portion of these samples. One more suggestion is to test such ensembles on labelled samples from different initial contexts: this may help to find which contexts are most similar to the target context.

(4)* Stacked ensembles* augment a pool of the base classifiers with a pool of their selection and combination strategies. Often, the choice of the selection or combination strategy depends on the training data size and may require data of target context only. The TV recommender in [90] split the data into different time windows and used two decision-making strategies on top of the base classifiers: the members' outputs were combined by voting in the beginning of the time window, whereas in the end the best base classifier was selected. The mixed data ensembles can be also stacked. An ensemble of the two simplest knowledge transfer strategies for interface adaptation in [31] was used when the target context data contained less than 15 samples, more intelligent strategies being added when the dataset size increased. This stacking was used for incremental learning of a new context because too small datasets make selections of a winner unreliable if the ensemble includes many strategies: the required amount of the training data depends on the ensemble size.

(5)* Dynamic selection* of a base classifier can be used in both context-specific and mixed data ensembles. Unlike “static selection” of the most accurate classifier for all data samples, the “dynamic selection” chooses for each test sample a base classifier that achieved the highest accuracy for the similar data samples. The conventional dynamic selection is based on estimating the similarity between test and training samples using their input features [95], as, for example, for the adaptation to different noise conditions in [96]. To model buying behaviour, the best ensemble member is selected in [97] for each product based on its features, such as promotions and dependency of sales on season.

Because the input features similarity is not necessarily preserved across different contexts, a nonconventional dynamic selection approach, proposed for adapting HMM-based systems in [98], is to compute output scores of all ensemble members for a test sample and to compare these scores with the training samples' scores. The ensemble is trained incrementally by adding new classifiers to and removing the least frequently used ones from a pool. However, learning how to select the best subset for each sample requires a 20–25% larger training dataset, comparing with the data needed for training the base classifiers. The overall need in training data depends on the ensemble size and task complexity.

Two other nonconventional approaches, proposed in [57, 68], select either the most appropriate relevance feedback technique for each query and image class by reinforcement learning in an ensemble of such techniques or the most appropriate classifier to handle missing data in affect recognition with a cascade of classifiers, respectively. In the latter case, social rules or personal differences in expressing emotions may exclude certain behavioural cues; for example, needs or habits to be silent result in the absent audio cues. To take the missing data into account, accuracies of all the classifiers are evaluated on training data and stored for each class: the most accurate classifier becomes a “specialist” for this class; the next by accuracy classifier becomes a “second best specialist” for this class, and so forth. The classes are ordered from the most to the least difficult for classification. At the fusion stage a sample is sent initially to the specialist for the most difficult class. If this specialist has classified the sample, the process is terminated to prevent classifying too many samples into the dominant class. Otherwise, the sample is sent to the next specialists until it is classified. To handle the missing data, the sample is sent to the “second best specialist” if the “specialist” for the corresponding class requires the missing modality.

### 4.4. Context as a Feature

Single and multiple classifiers, using context cues as latent factors, network nodes, or input features, constitute the “*context as a feature*” group. This approach is most feasible in cases when the context cues are discriminative features. The second and third cases also require automatic context recognition. Lazy methods often include context descriptors in a similarity measure. The adaptation can be to either exactly the same context factors as included into the classification model or higher-level situations, described by a set of fine-grain parameters. The adaptation to social rules in different groups using group members' identities or roles as features exemplifies the latter case. Context cues used inside the model increase its complexity and thus the need in training data. Data collection efforts depend also on whether training datasets are context-specific or mixed.

- *Context-specific models* are trained on the target context data and suit well cases when user and system behaviour depend on certain fine context parameters, but the dependencies vary for different rough situational strata: for example, dependency of buying behaviour on time of the year differs in different cultures. Therefore, the time context can be a feature, but learning separate models for coarse situations, such as different cultures, is more feasible.
- *Mixed data models* are trained on the data from the target and other contexts or obtained by modifying parameters of models for the initial context. These models are often used for adapting to exactly the same context factors as included in the model. Training a single model for fairly broad ranges of context values allows consistent modelling of context dependency, but accommodating the previously unseen context values would require complete retraining.

Training these models may use either the approaches in Section 4.2 or described below additional four ones, which can be applied to both the context-specific and the mixed data models (the required amounts of training data depend in these cases predominantly on the model complexity).

(1)* Embedding contextual parameters as additional nodes* into graphical models is used in affect recognisers, recommenders, and other systems. A dynamic Bayesian network for drivers' emotion recognition in [99] included additional nodes, taking predefined discrete values to represent both the environmental context (complex versus simple road situation) and the user characteristics (skills, physical condition, and mental state). This allowed to interpret the video cues (e.g., high versus low eyelid position and high versus low gaze fixation) and audio cues (answers to questions) in a context- and user-dependent manner. Data collection was avoided by specifying network parameters by hand. For photo annotation, such nodes can be time, location, and camera parameters, for example, flash [100] or clothing detection, presence of other persons in photos, and demographic statistics for estimating a probability that a person of a certain age and gender has a certain name [101]. In the recommenders the context factors are used also as latent variables, for example, predefined purchase goals as a latent cue in Bayesian networks [102].

(2)* Using historical contexts as nodes in graphical models*: classification of a current data portion in multimedia analysis may depend on the classification results for the previous portions. For example, replays in sport videos usually follow goals but not vice versa. This kind of temporal context can be modelled by HMM, Bayesian networks, or correlation-based graphs [103–105]. Similarly, in affect recognition tasks the past emotional states are often used as nodes, for example, in HMM [75, 106] and so-called Long Short-Term Memory neural networks (LSTM) [106, 107].

(3)* Using contextual parameters as input features*, for example, in the support vector machine (SVM), case-based reasoning (CBR), multilayer perceptron (MLP), naïve Bayes (NB), and decision tree (DT) classifiers: in affective computing a past emotional state served as an additional input to the SVM [75, 108] and AdaBoost [109]. The emotion recogniser for a spoken dialog system [110] employed as the context predefined past events, such as dialogue acts (e.g., repetition and rephrasing, needed when a user cannot be understood immediately) and lexical expressions (e.g., the user's words “no, I said…” may imply correcting a system's mistake that does not make the user happier). Probabilities of emotional categories, associated with these events, were combined with probabilities estimated from acoustic and prosodic features in two stages, employing each a nontrainable fusion, for example, the voting, average, or product of the probabilities. Similarly, dialog acts served as context for differentiating between “doubtful” and “bored” user states in [111].

Time and other predefined fine context descriptors can serve as features in the TV recommenders, learning from long-term interaction histories, but the recommendations are actually adapted to indefinable coarse situations, such as differences between the personal and family cultures. Recommendations for individuals in [112] used a day of week, time of day, user location, and device contexts as inputs to the CBR, MLP, NB, and DT. Recommendations for families in [52, 113] used time and personalities of family members as inputs to the SVM and CBR. Although the “presence of family members” was here a predefined context type, sets of family members were not predefined and varied between the families, which were of different sizes and with or without children. Such learning of the group preferences by observing choices, made by the group members together and separately, respects group practices rather than enforces a practice chosen by a system designer, as was done, for example, in [88] (described in Section 4.2.4). This approach requires no domain knowledge either, but it needs data: for example, achieving reasonable recommendation accuracies in [113] required training data collection during nearly one month. A way to shorten data collection time is to adapt to selected characteristics of users instead of their identities: for example, in [53] “big five” personality traits of individuals served as input features to a neural network, trained to adapt interaction style (e.g., dialog-based and browsing). This approach requires additional data to obtain personality traits, though: in [53] these traits were obtained via analysis of posts, written by the test subjects in social networks.

(4)* Including contextual similarity into a distance measure* is common for context modelling in the CF-based recommenders: for example, distances between the users' ratings can be weighed by similarities between predefined contexts (utilitarian versus hedonic users' needs, a day of week, and time of day), in which these ratings were provided [114]. Including context in a distance measure can also help to deal with changes in the users' interests over time: for example, an order of items' consumption and difference between consumption times [115].

### 4.5. Comparing the Adaptation Approaches

Experiments with more than one adaptation framework are reviewed in brief below. Unfortunately, no consistent experimental comparisons of the different approaches could be found in the literature. Moreover, while in some cases only the lightweight adaptation led to the desired functionality [31], the fine adaptation required in other cases so significant data collection efforts that even the application developers did not want to meet with these problems [75].

The adaptation is more lightweight if feature selection is not performed for each context. Using the same features was compared with the feature selection in [116] for object categorisation by fully supervised learning vector quantisation method. The data were challenging as it contained images with rotated by various angles objects of different shapes and colours. Using the same features for different object categories did not notably lower the accuracy, comparing with the feature selection. Therefore, a good initial choice of the features may allow for learning new categories even without extensive feature selection for each new data portion.

In other comparative experiments, discussed below, feature selection was not performed for each context. The ensemble-based adaptation was compared in [37, 81] with three methods to build context-specific SVMs for detecting concepts in the TV data. The SVMs, trained separately on the data from different contexts and combined using a weighed sum with weights stating importance of the new context, formed the ensemble. The context-specific SVMs were built in the following ways: (i) by the model-level knowledge transfer (described in Section 4.2.2); (ii) by training on the merged data from the old and new contexts, and (iii) by training on the data of the target context only.

In the tests on the data from 13 TV channels, the model-level knowledge transfer was close by the average accuracy to the models, trained on the merged data, but the training was up to 15 times faster. The purely context-specific models were less accurate due to a small number (from one to ten per concept) of positive training samples. The ensemble accuracy was close to that of the knowledge transfer if the target context weight was not high. Additionally, a semisupervised SVM, trained on both the labelled and the unlabelled target context data, but with no knowledge of initial contexts, was evaluated in [37] and appeared to be significantly less accurate than the model-level knowledge transfer.

Several ways to build a context-specific HMM for recognising excitement of show audience from an audio-visual stream were compared in [75] (these ways are described in Sections 4.2.1 and 4.2.2): (i) cascaded training on the target context data; (ii) model-level knowledge transfer (either the context-specific HMM, trained for some other context, or a general HMM was adapted to the target context, using its labelled data), and (iii) unsupervised training of the conventional HMM on the unlabelled target context data. To demonstrate benefits of the context adaptation, the context-specific models were also compared with a general HMM, built either by unsupervised or by cascaded training on the data of all contexts.

The cascaded training on the target context gave the highest accuracy in these tests. The general HMM, trained in the cascaded way, was also more accurate than the conventional HMM. Although feasibility of unsupervised pretraining of deep neural networks was demonstrated in [74], in training of less deep architectures usually all the available data is utilised at once. According to [26, 75], the cascaded training may be beneficial in nondeep architectures, too.

The model-level knowledge transfer achieved slightly lower accuracy than the cascaded training. As regarding the choice of a model to modify, various initial context models were quite similar by their accuracies after adapting to the target contexts (probably, due to the notably different contexts). The adapted general model was slightly more accurate. The conventional context-specific and general HMMs achieved significantly lower accuracies than the model-level knowledge transfer. In addition, accuracy of fully supervised SVMs, trained on the labelled target context data, was also presented in [75]. In the tests the SVM and HMM accuracies were similar to each other for a large number of the annotated samples provided, but with only 25 labelled samples per context the SVM became much less accurate than the HMM.

The cascaded architectures were beneficial in other applications, too. Updates of final rather than initial stages of a cascaded system in [32] were more efficient for speech processing. A single model, trained for the TV recommender in [55] on several attributes of TV programmes, adapted to individual tastes less accurately than a classifier ensemble. Each member of the latter was trained on a single programme attribute (such as “genre”), and the models' outputs got different weights for the different users. The hierarchical and nonhierarchical methods of learning interconcept relations to detect concepts in images were compared in [76, 103]. The hierarchical approach built first binary classifiers for each concept separately and then adapted their relations to contexts [76] (more details are provided in Section 4.2.1), while the nonhierarchical learning built such a relational model directly from the low-level features [103]. In the tests both approaches achieved fairly similar accuracies, but the hierarchical adaptation was significantly faster.

Different ways of knowledge transfer have been compared in a study into pain recognition ([92] (described in Section 4.3.2)). The lightweight adaptation of a combination-based ensemble was compared with two approaches: (1) training of a person-specific model using data of the target person only and (2) training of a person-specific model using data of all test subjects. The tests proved that lightweight adaptation is notably faster: it took 0.16 minutes per person on average, while training on the data of the target person took 2.6 minutes and training on the data of all subjects took 14.3 minutes per subject on average. The ensemble was also notably more accurate than a person-specific model, trained on data of the target person only, in cases when the number of labelled training samples per target person was fairly small (from 10 to 50 samples). The accuracies of the ensemble and of the person-specific model, trained on data of all subjects, were fairly similar when number of labelled training samples per target person ranged from 10 to 25, but the ensemble was notably more accurate when training dataset per target person included 50 and more samples.

Different knowledge transfer strategies have been compared in [31, 84] in a study into user interface adaptation. The ensemble of several simple strategies was used in [31] (described in Section 4.3.2), and two popular alternative approaches were tested in [84] on a part of the dataset used in [31]. One of them transfers the knowledge by adding or subtracting a shift vector from the vectors of preferences for each user (or user group), similarly to [47]. Comparing to other approaches, its accuracy in the tests was considerably lower. The second approach exploited one of the most common adaptation methods: modifying a similarity measure by an optimisation algorithm to minimise the prediction error for all nontarget users. This approach appeared to be fairly accurate and outperformed other approaches in cases when the users' preferences in the initial context were similar to each other but differed significantly in the target context. However, this approach required much longer computations, and its average accuracy was similar to that of the ensemble in [31].

The use of the target context data only versus the mixed data in the ensemble of relevance feedback strategies for image retrieval was explored in [68] (described in Section 4.3.3.). The target context included interactions of a current query, and the mixed data contained interactions of multiple retrieval sessions of multiple users. In the tests the mixed data greatly increased the precision of initial results (images retrieved before using the relevance feedback). The precision after the first relevance feedback iteration was also improved, but less notably, whereas after the second iteration the precision with the mixed data was only 2.5% higher than that with only the current query data, and the time gain was insignificant, too.

Adaptation to uncontrolled environments has to deal with possibilities that not all input modalities are always available. The cases of missing or poor quality data samples were studied in biometrics, because an important goal for next generation biometrics is to increase user convenience [117, 118]: for example, users may dislike certain biometric modalities or be incapable of providing data due to trauma. The missing data can be handled by training models for different combinations of modalities and selecting an appropriate model for each combination [119, 120] or by applying generic methods to fuse all modalities in presence of missing data, such as imputation of the missing samples or modification of the fusion algorithm [121]. Suitability of generic methods, however, depends on missingness mechanism, that is, whether data are missing at random or not [121]. For example, imputation methods do not fit well the “data are missing not at random” scenario [121], whereas “data are missing at random” assumption is infeasible when context influences availability of modalities (e.g., if etiquette requires silence, voice data will be unavailable). Besides, studies, comparing the model selection with imputation and the modified SVM fusion [119, 122], demonstrated that the model selection was more accurate on the average. Samples of various qualities in biometric systems can be handled also by model selection (training aseparate model for each quality cluster) or using the sample quality as an additional input feature. According to the tests in [123], the former approach is more accurate, especially if the number of the quality measures increases. These results suggest that non-discriminative contextual parameters, like the sample quality factor, should not be used as features.

Fine context descriptors, such as time, can be discriminative factors in the recommender systems. Use of context descriptors as features in the trained (SVM) and lazy (CBR) classifiers in the TV recommenders was compared in [52, 113] (methods are described in Section 4.4). In the tests on real-life TV viewing histories of 20 families both classifiers achieved quite similar average accuracies.

Table 1 presents most important characteristics of the reviewed lightweight adaptation approaches. These features can have positive or negative influence on adaptation, depending on the task at hand. For example, ability of the adaptation approach to use unlabelled data for the target context is a positive feature because it decreases the need in the labelled data. On the other hand, such approaches cannot be employed in applications where unlabelled data cannot be obtained. Similarly, most lightweight approaches suit well cases when only very little datasets for the target context can be obtained, but their accuracies are usually lower than that of the less lightweight methods. (“Most lightweight” and other characteristics of adaptation approaches in Table 1 refer to runtime training in case when a new context emerges.)

## 5. Lightweight Adaptation Design

The above overview allows us to identify important application characteristics, influencing the adaptation design, and suggest choices of the adaptation methods, which are most likely to work for different application requirements.

### 5.1. Choices and Factors Influencing Design Decisions

Let the same types of data and context cues, although not necessarily exactly the same sets of cues, be used in all contexts. Then the most important design choices are the interaction, adaptation, and data usage types. The interaction can be implicit or explicit or can combine both. The adaptation types are model selection, classifier ensemble, or using context as a feature. The data usage type is the knowledge transfer type and level: either no data, or a dataset, acquired in other situations, or a model trained on a dataset, acquired in other situations, can be employed.

These choices strongly influence choice of a reasoning method and choice of runtime training and supervision types. The reasoning method can be either lazy or trained graph-based (e.g., HMMs and Bayesian networks) or non-graph-based (e.g., neural networks and SVMs). Runtime training can be performed by either conventional standard algorithms (e.g., the Baum-Welch one to train HMMs) or custom parameter modifications, including the choice of parameters to modify and/or certain simplifying assumptions, for example, linearization of functions and constraints on parameters' changes. The supervision can be full or partial (unsupervised context adaptation usually ignores peculiarities). All these decisions depend on each other and on task specifics.

The following application characteristics influence the design the most:

- *Expected changes in input cues in different situations, namely, in their (1) meaning; (2) availability; (3) influence; and (4) accuracy*: for example, availability of all cues is not guaranteed in uncontrolled environments; the low-level cues, for example, image features, usually have no meaning to change, whereas high-level behavioural cues may easily change meaning, for example, user's silence after a show is likely to indicate user's disappointment as satisfied users would applaud, whereas silence in the beginning is more likely to indicate neutral mood (just waiting for a show to start).
- *Effects of different interaction types (i.e., costs of data acquisition versus data quality): (1) implicit interaction and (2) explicit interaction*, that is, whether users and classifiers can significantly benefit from explicit human efforts and whether implicit interaction can be reliably interpreted.
- *Variability of situations a system is likely to encounter: (1) stable versus unstable and (2) definable versus undefinable situations*: for example, an in-house TV programme recommender should adapt to any family acquiring it, but only to this particular family, whereas an Internet TV programme recommender service has to adapt to each family using the service. To define several screen types for adapting an UI is an easy task but to specify all situations where humans' behaviour is governed by social rules is much more difficult. Such elusive situations will be called “indefinable” below.
- *Adaptation time*: that is, whether the adaptation must be very quick (e.g., the interface should be adapted just at the moment of launching an application) or can take time or whether a lifelong learning is expected.

**Figure 3:**
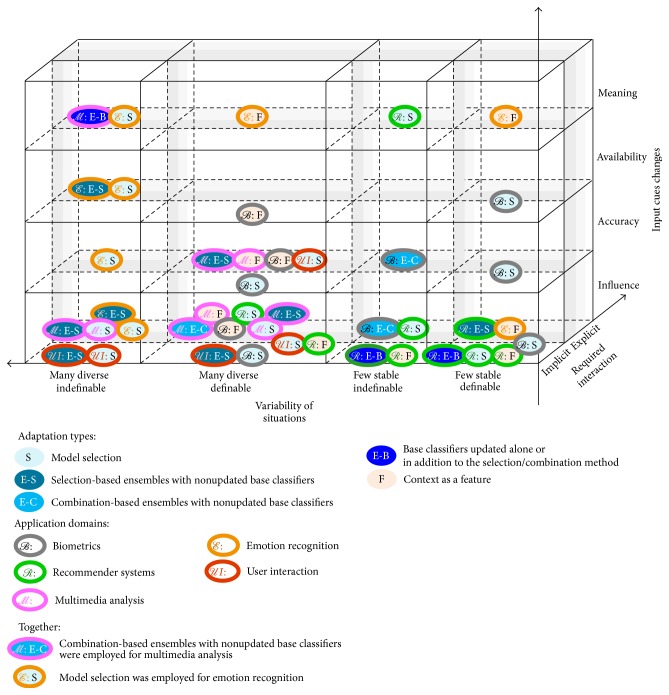
Lightweight adaptation summary (Section 4).

Figure 3 shows which adaptation approaches, outlined in Section 4, were suggested for different subtypes of the three most influencing types of application characteristics. Although many of these approaches were used offline, the proposed techniques suit the runtime adaptation, too. Subtypes of the application characteristics are ordered along the corresponding axes by increasing complexity: among changes in input cues, meaning changes are the trickiest, while the simpler changes in the cues' influences are the most common case; among interaction types, the explicit interaction is considered more difficult than the implicit one because the former often results in very small datasets. Among situations types, “many diverse indefinable situations” is the most difficult case. The application characteristics are either taken from the papers reviewed or based on our own assumptions: for example, the accuracy and meaning of low-level image features, such as colour histograms, rarely depend on context. In particular, if a colour histogram is used for distinguishing between cars and houses, its meaning would be database-dependent only if the majority of cars and the majority of houses are red and green, respectively, in one database, while these colours are swapped in another database. However, this is too unlikely in the large databases. Therefore, we assume that the low-level image features can only change their influence on a classification result.

### 5.2. Interaction Types

As shown in Figure 3, the adaptation to changing input cues' meanings is rarely addressed. Several studies suggest that quick learning of new meanings may be difficult without explicit human supervision. Thus, acquiring explicit interaction data can be suggested for the changes in meanings of the input cues. Explicit efforts are necessary also if interpretation of implicit interaction data depends on context: for example, if just the same user actions may denote a positive feedback in one context and neutrality in another context.

The implicit interaction should be employed, if possible, when quality of primary data cues is rather low. For example, if a certain cue is important for users, but not recognised by a system, accurate models cannot be built despite all the explicit annotation efforts; instead, the latter will only annoy the users. Due to this reason, TV recommenders often employ an implicit feedback: TV programme metadata is rarely detailed.

Otherwise, for choosing between the implicit and explicit interaction we recommend considering, first, how the UI design affects quality of implicit data and, second, to what extent both labelling time and a time interval when the labels are used by the system match the users' goals. For example, if the users click on nearly every link because of insufficient link data, the implicit feedback would be useless. On the other hand, benefits of explicit feedback should last longer than its acquisition: for example, interactive multimedia retrieval systems usually learn from small datasets because users only see how the current query results are improved by their feedback, whereas recommender systems may require users to rank many items and to use these ranks for next year(s). Third, we recommend considering whether users' actions follow their own desires or social rules: in the latter case the implicit feedback may be useless if the users adjust their choices to be polite. This behaviour, however, is less common among loving families and close friends than in groups where some members dominate. To understand whether all group members are equally satisfied is difficult [20, 31], but solutions to this problem remain to be found.

### 5.3. Adaptation and Data Usage Types

**Figure 4:**
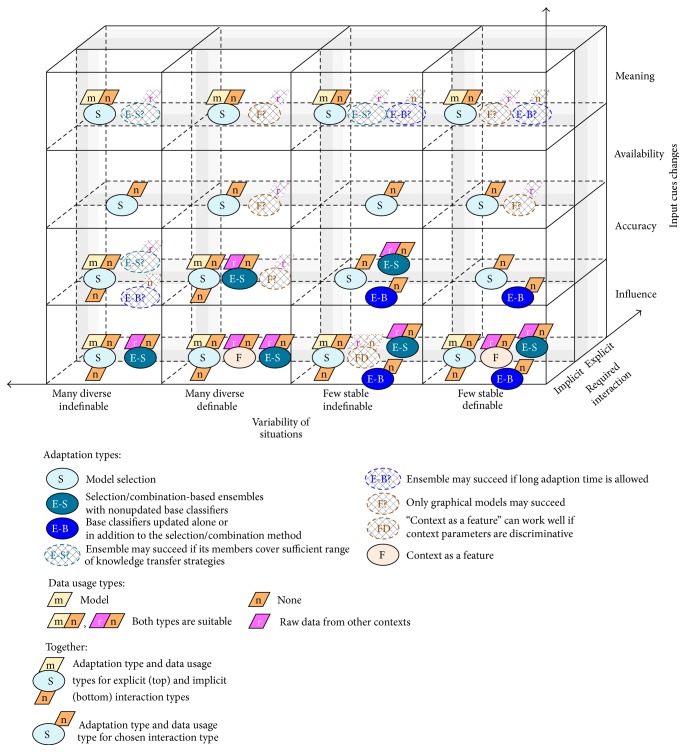
Recommended adaptation and data usage types.

A very quick adaptation can be performed by (i) selecting from a set of models, trained for predefined situations; (ii) using the context as a feature in a model, trained for a broad range of context cues, and (iii) employing selection-based classifier ensembles with nonadaptive base classifiers. Recommendations for choosing adaptation and data usage types for other cases are given in Figure 4. These choices are closely related, but the usages of data of one or several nontarget situations are not differentiated because no sound clues exist in the state-of-the-art research.

Using both labelled and unlabelled target context data could be beneficial when both could be obtained. Benefits of using unlabelled data of the nontarget situations in the lightweight adaptation have not yet been demonstrated. Usually, the decision on whether to use only the target context data or also the labelled data from other situations for adapting to a target context is based on the domain knowledge or data comparisons for these situations (e.g., comparison of data distributions). The latter approach is not quite lightweight, so we suggest employing simple heuristics instead: the more serious the changes in the input cues are expected, the more dissimilar the situations in question are.

As Figure 3 shows, cases of changes in influence of input cues on adaptation result are best studied. If just such changes are expected and an application will be used in a few fairly stable easy-to-define situations, nearly any adaptation approach from Section 4 can be applied. In cases of changes in meanings of input cues choice of adaptation approaches is rather limited. Training a separate model for each context is one of the most adaptive approaches. It can be applied to a broader range of cases than those having been studied so far and handle primary data cues, emerging at runtime, provided that their types and the use of different types inside the models can be predefined and the models can be trained at runtime. On the other hand, the adaptation to changes in the input cues availability may be reached without training a separate model for each context if the cue availability is treated as a feature in reasoning methods that handle the missing cues without retraining, for example, discrete HMMs, weighted sums, and voting.

Among the above-mentioned approaches to obtain the context-specific models, two generic ones can be emphasised: (i) the* cascaded training* using only the target context data if the unlabelled data is available and (ii) the* model parameters' modification by evolutionary algorithms*. Cascaded training was proved beneficial for deep neural networks [74] and MLP [26] (trained conventionally in fully supervised ways) as well as for HMMs (trained conventionally in unsupervised ways) [75]. Evolutionary algorithms are worth considering because they are fast and applicable to various sets of parameters. Furthermore, they require no differentiable penalty function and are not easily stopped by local minima. These algorithms can be employed for both the model-level knowledge transfer (shifting a decision boundary) and the cascaded training (at its last stage). Other learnable models may also benefit from the evolutionary adaptation.

The classifier ensemble, where all members are trained on the target context data, is the most adaptive but is less lightweight than training a separate model for each context. Accordingly, employing such ensembles to adapt to contexts, emerging at runtime, is feasible mainly when large datasets are acquired naturally in the course of using an application, for example, when implicit interaction data can be collected. In other cases, either classifiers learnable from very small datasets are to be included into the ensembles or other ensemble types should be chosen.

Combining outputs of the base classifiers can be recommended only when all these classifiers are sufficiently generic (e.g., in factor ensembles) or trained on target context data. In other cases the selection-based ensembles should be employed: they can handle both similar and dissimilar contexts if different members are optimised for different contexts [31], and they outperform individual classifiers when amount of training data is not abundant [95].

Unlike the conventional classifier ensembles, built from pattern recognition strategies, the context-adaptive ensembles may include other reasoning strategies. Among the latter, the knowledge transfer strategies are most suitable for cold-start adaptation to newly emerged contexts. Such ensembles can adapt to new contexts without retraining the base classifiers after only a few target context data samples are acquired, provided that the transfer strategies can be defined for a task at hand. On the other hand, the ensemble should not be of too large size because the more the members to be evaluated, the more the data required [31]. The ensembles can also handle the primary data cues, emerging at runtime, if some of their members (e.g., lazy methods) handle such cases. Typical behaviours of individuals and groups [31]; typical weights of input cues; and typical types of relations between input or output cues (yet to be tested) may be included into the knowledge transfer strategies' ensembles. After more target context data is collected, more sophisticated adaptation methods can be employed instead, for example, by stacking the ensembles.

Recommendations on using the context as a feature refer to single classifiers. Single classifiers handle changes in the input cues' influences the best. The graphical models, for example, HMMs and Bayesian networks, adapt to such changes by adjusting the observational or transitional probabilities. The HMMs suit well adaptation to the historical context factors. Using context parameters as features in nongraphical models (e.g., in the SVMs) can be recommended only when the context factors are discriminative. Similarly, model selection and ensembles can also include models, using context descriptors as features.

Regarding the data usage types, training the context-specific models on the merged data of different contexts may hinder the adaptation to notably different contexts. Therefore, the use of the merged data can be recommended only if the chosen adaptation requires such data: for example, for an ensemble of knowledge transfer strategies or for optimising similarity measures. Using trained models of the nontarget contexts can be recommended for faster adaptation. To use the target context data only can be recommended when the training datasets are not too small (e.g., if implicit interaction data or unlabelled data are available) or the applications are supposed to be long-term user companions and thus need to gain the user's trust by avoiding data sharing and reasoning errors.

### 5.4. Training and Supervision Types

Choices of the training and adaptation types are strongly interdependent. Model-level knowledge transfer and old knowledge preservation usually require custom algorithms; for example, some additional constraints on the model parameters may be added. Custom adaptation, such as with evolutionary algorithms or various reweighting schemes, is usual in selecting and/or combining the ensemble members, too. Standard training is more common in other cases due to its easiness for developers.

The choice of a training supervision type depends strongly on the chosen interaction and data usage types. If no knowledge transfer is used, the training dataset, especially the one obtained via explicit interaction, may be too small for the fully supervised training. If an application allows for acquiring the unlabelled data, the cascaded training should be employed. The conventional semisupervised training cannot be recommended for adapting to contexts, emerging at runtime, as correct modelling assumptions are difficult to make for new contexts and the incorrect ones decrease the accuracy of the semisupervised learning comparing with the use of the labelled data only [26].

## 6. Conclusions

The lightweight runtime adaptation of classifiers to newly emerging situations, not requiring significant explicit interaction efforts and the detailed domain knowledge, is a new research area with important practical applications for user-centric multimedia analysis and retrieval, automated UI adaptation, recommender systems, and so forth. We reviewed promising adaptation techniques, proposed in several application domains, and provided recommendations on selecting such techniques, based on the identified application characteristics and simple heuristic evaluations of similarities between contexts. Our focus is on adapting class-level and decision-level multimodal fusion under the assumption that inputs to the fusion models (called cues) are provided by the same algorithms in all contexts. Several studies have shown that despite this significant limitation the lightweight adaptation can be close by classification accuracy to more computationally expensive adaptation methods, whereas in many cases it simply has no alternatives to compare with. Moreover, if the full-scale adaptation is possible, it can be performed after the lightweight one, either if the end users did not accept the latter results or when additional data becomes available.

Two main problems of the full-scale adaptation are the extensive use of the domain knowledge in reasoning and the need in the labelled training data. The above lightweight approaches are more data-driven than domain knowledge dependent; that is, they employ sufficiently generic input cues and learn to handle them in context-specific ways, using the target context data. For example, the same user or system behaviour can be assigned to different inner states of a HMM classifier by modifying observational probabilities (in particular, the classifier can be trained to treat a sound of “whistling” as a sign of the user excitement in one context and “nothing special” in another context). Ensembles of methods for knowledge transfer between contexts and data of several contexts allow for learning which transfer method is more appropriate for the initial and target contexts at hand: for example, which relevance feedback strategy better suits the current user query or which types of user preferences strongly depend on contexts. The domain knowledge dependency can be reduced also by employing the reasoning that naturally deals with missing inputs and cues emerging at runtime. For example, graphical models can treat a missing cue as a valid observation; if various types of cues are read in different ways from data logs, it is sufficient to specify how to treat a cue type in reasoning and specifying an exact set of the cues is unnecessary.

At the same time, the need in the labelled data can be reduced by using the unlabelled data in addition to the labelled data; by modifying training algorithms so that very small sets of the labelled data would be sufficient; or by reusing the knowledge regarding multiple contexts, or multiple users. For example, the conventional assumption that users, similar to each other in the past, remain similar also in the current situation does not necessarily hold for significantly different past and current situations. Therefore, the user community data can be used first to check whether users, similar to each other in a certain initial context, remain similar also in a target context. Then, if this would be the case, the data for this initial context can be used in addition to the data of the target context. Otherwise, only target context data should be used.

That the lightweight adaptation is feasible was confirmed in the aforementioned studies. However, trade-offs between the adaptation cost and performance gain, acceptable for the end users, remain an open problem. Moreover, the user acceptance depends not only on the classification accuracy but also on many other factors, such as user companions, screen sizes of interaction devices, general user attitudes to adaptability of personal devices, and reasoning implementation. For example, in the UI adaptation [31] the reasoning employed user community data, and many subjects preferred this way to a more common launching of the applications with settings provided by application designers, because of the higher confidence in their acquaintances. Thus, certain types of knowledge transfer, in addition to reducing the need in data collection for the target situation, may also facilitate the users' trust.

The lightweight adaptation is suitable for various practical problems, including a cold-start adaptation when it is not yet possible to acquire sufficient data for more sophisticated methods. Also, it is beneficial for interactive systems that should respond fast to users' requests, as well as for systems encountering a large variety of the usage situations. The designers of the latter systems would be unable while the end users will be unwilling to invest big efforts in data collection. A full-scale adaptation should not be the only option for the end users of both kinds of systems; it may be more feasible to try the lightweight one first to see whether the users are satisfied with it. Although the lightweight adaptation methods, reviewed in this work, were usually tested on the data of one application domain only, we believe that other domains can also benefit from these studies. For example, the work [2] concluded that pervasive computing applications should employ knowledge transfer learning methods to greater extent to reduce data collection needs. Among the lightweight adaptation methods reviewed one can also find generic enough ones, being useful not only for context adaptation but also for user adaptation and solving other machine learning problems.

## Figures and Tables

**Table 1:** Characteristics of the reviewed adaptation approaches. Notations: X: yes; A: depending on a chosen algorithm; for example, training data can be used instead of assumptions about new context and unlabelled data are used only as negative examples; MK: multiclass classification problems only; U: data are vectors of user preferences; base classifier: either ensemble member or the only context-specific model.

Method name	Applicability				Training		Additional data			
	Suits dissimilar contexts	Suits large varieties of problems/algorithms	Most lightweight	Requires little assumptions about new contexts	For training of base classifier(s)	For classifier selection/combination	Uses contextual data	Uses raw primary data of other contexts	Uses models for other contexts	Uses unlabelled data for the target context
Model selection										
Contextual weighting	X	X	A	A	X	X	A			
Optimising utility function	X				A	A	A			A
Tuning classifiers for small datasets	X			X	X					A
Cascaded training	X	X		X	X					X
Learning context-specific relations between classifier outputs	X	MK	X	X		X				A
Optimising model parameters with evolutionary algorithms	X	X		X	X	X		A	X	
Optimising model parameters with gradient descent	A	X		A	X			A	X	
Algorithm-specific methods to shift a decision boundary	A			X	X			A	X	A
Adapting only selected parameters	X	X	X		X	X		A	X	
Error weighting		X			X	X		X	A	
The use of model parameters as training data	A				X				X	
Vector modification	X	U	X	X	X			X		A
Modifying a similarity measure	X	U	A	A	X			A	A	A
Target context-specific combinations of cues, obtained in other contexts	X	U	A		X	X	A	A		A

Ensembles										
Factor ensembles	X		X	X		X				A
Diversity-based ensembles	X	X		X	X					A
Optimising a pool of classifiers, trained on data for several contexts		X	X	X		X			X	
Ensemble of generalisers			X			X	X		X	
Knowledge transfer ensembles	X	X	X	X		X	A	X	A	A
Stacked ensembles	X	X	X			X				
Dynamic selection of base classifiers	X	X	A	X		X	A	A		
Sample-selecting ensembles	X	X		A	X			X	A	A

Context as a feature										
Embedding contextual parameters as additional nodes into graphical models	X				X		X	X		A
Using historical contexts as nodes in graphical models	X				X		A	X		A
Using contextual parameters as input features	X	X		X	X	X	X	X		A
Including contextual similarity into a distance measure	X		X			X	X	X		A
